# From project aid to sustainable HIV services: a case study from Zambia

**DOI:** 10.1186/1758-2652-13-19

**Published:** 2010-06-07

**Authors:** Kwasi Torpey, Lona Mwenda, Catherine Thompson, Edgar Wamuwi, Wim van Damme

**Affiliations:** 1 Family Health International/Zambia Prevention, Care and Treatment Partnership, Lusaka, Zambia; 2 Ministry of Health, Lusaka, Zambia; 3 Institute of Tropical Medicine, Antwerp, Belgium

## Abstract

**Introduction:**

Sustainable service delivery is a major challenge in the HIV response that is often not adequately addressed in project implementation. Sustainable strategies must be built into project design and implementation to enable HIV efforts to continue long after donor-supported projects are completed.

**Case description:**

This paper presents the experiences in operational sustainability of Family Health International's Zambia Prevention, Care and Treatment Partnership in Zambia, which is supported by the US President's Emergency Plan for AIDS Relief through United States Agency for International Development (October 2004 to September 2009). The partnership worked with Zambia's Ministry of Health to scale up HIV clinical services in five of the country's nine provinces, reaching 35 districts and 219 facilities. It provided technical and financial support from within the ministry's systems and structures. By completion of the project, 10 of the 35 districts had graduated beyond receiving ongoing technical support.

**Discussion and evaluation:**

By working within the ministry's policies, structures and systems, the partnership was able to increase the ministry's capacity to add a comprehensive HIV service delivery component to its health services. Ministry structures were improved through renovations of health facilities, training of healthcare workers, procurement of essential equipment, and establishment of a quality assurance plan to ensure continued quality of care. The quality assurance tools were implemented by both the ministry and project staff as the foundation for technical graduation. Facilities that met all the quality criteria for more than six months were graduated from project technical support, as were districts where most supported facilities met the criteria. The district health offices then provided ongoing supervision of services. This predetermined "graduation" exit strategy, with buy in of the provincial and district health offices, set the stage for continued delivery of high-quality HIV services.

**Conclusions:**

Achieving operational sustainability in a resource-limited setting is feasible. Developing and institutionalizing a quality assurance/quality improvement system is the basis on which facilities and districts can move beyond project support and, therefore, sustain services. Quality assurance/quality improvement tools should be based on national standards, and project implementation should use and improve existing health system structures.

## Introduction

### Sustainability in the global context

Universal access to prevention and treatment for all [1,2] is an integral part of the global agenda to mitigate the HIV pandemic. Sustainable strategies must be built into project design and implementation to enable HIV efforts to continue after the project has run its course, particularly in countries highly dependent on donor funds [3,4]. The lack of sustainable project implementation contributes to the unmet need for comprehensive HIV services in resource-limited countries beyond rapid scale up and expansion [5-7].

### Concept

Sustainability of health programmes and services can be defined as: "... the capacity to maintain programme services at a level that will provide ongoing prevention and treatment for a health problem after termination of major financial, managerial and technological assistance from an external donor" [8].

It is further defined as "how well programmes become institutionalized in organizations or health and social systems" [9].

### Scope of article

This paper presents operational sustainability experiences from Family Health International's (FHI's) Zambia Prevention, Care and Treatment Partnership (ZPCT), supported by the US President's Emergency Plan for AIDS Relief (PEPFAR) through the United States Agency for International Development (USAID). It focuses on an approach to facilitate technical and programmatic sustainability, which are key components of operational sustainability in implementing HIV clinical services in Ministry of Health (MOH) facilities.

ZPCT worked with the MOH in Zambia to scale up HIV clinical services in 35 districts in five of the country's nine provinces. Selected health facilities were provided with technical and financial support, as outlined in a recipient agreement with a detailed scope of work. This agreement was executed within MOH systems and structures. Clinical HIV services included prevention of mother to child transmission (PMTCT) of HIV, counselling and testing, antiretroviral therapy (ART), clinical care, and HIV-related laboratory and pharmacy services, enhanced by information management systems.

### Elements of sustainability

Service sustainability is a complex concept that can be classified into four elements: technical, programmatic, social and financial sustainability.

*Technical sustainability *is the continuous provision of high-quality, facility-based HIV clinical services aligned with national standards. Service provision requires, at a minimum, effectively organized, trained human resources at service delivery points implementing the basic package of HIV prevention, care and treatment, as defined by national standards. This includes: adequate laboratory and pharmacy infrastructure; sufficient equipment and commodities; functional health management information systems; and a basic laboratory sample referral system to enable shared access to scarce laboratory and human resources across the healthcare system. Underpinning this are clear national guidelines and standard operating procedures on HIV service delivery, and a quality assurance/quality improvement (QA/QI) system to ensure that the standards are being met.

*Programmatic sustainability *entails effective management, coordination and implementation of facility-based HIV services. The provincial health offices and district health offices play a major role in facilitating this sustainability within Zambia's decentralized system. It requires establishment of: robust logistics; commodity and supply management systems; measures to ensure continuous service provision at facility level, uninterrupted by staff transfers or attrition; functional communications; transport; management of scarce resources; and a culture of evidence- and performance-based planning, utilizing the health management information systems, as well as quality improvement systems, to inform decision making. Stakeholders' access to information sharing and feedback is essential in the process.

*Social sustainability *refers to sustained HIV activities, which rely on continued demand for HIV services by communities [6,8]. Demand is enhanced by interventions deemed acceptable, accessible, affordable and culturally sensitive, with deliberate efforts to minimize stigma and maximize community awareness and mobilization.

*Financial sustainabilit*y involves adequate and continuous funding to achieve HIV service targets and objectives. This is a major challenge in resource-limited countries. The ability of governments to secure long-term health sector funding, either from internally generated resources or from donors, to gain financial sustainability for HIV services is a vital requirement [6,10]; this issue is beyond the scope of this paper.

**Figure 1:**
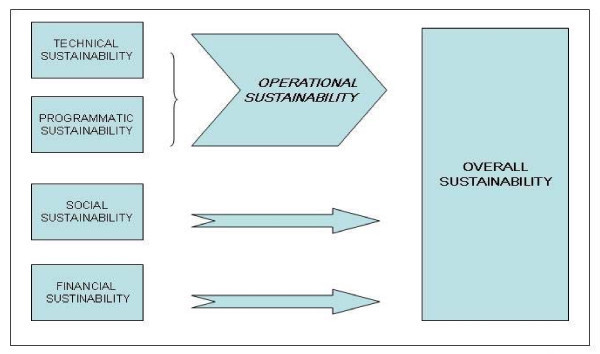
**Conceptual framework for overall sustainability of facility-based HIV services**.

Attaining overall sustainability of facility-based HIV services requires all four elements to be addressed (Figure 1). Employing strategies to achieve technical and programmatic sustainability would achieve operational sustainability of facility-based HIV services.

Necessary contextual factors include enabling national health policies, collaboration among non-governmental organizations and a conducive political climate from both government and donors [11]. Adoption and enforcement of the "Three Ones" principles by government and donors, namely one national action framework, one coordinating authority and one monitoring and evaluation system for HIV, creates a favourable atmosphere for coordinating sustainable HIV responses [12]. A decentralized health system, with the MOH linked to management units at provincial, district and health facility levels through which the HIV response can be monitored and delivered is essential. It allows effective collaboration with project staff at similar organizational levels.

The fragile state of the healthcare system creates a challenging environment that affects the MOH's technical, programmatic and financial efforts to contribute towards building long-term sustainable HIV interventions [4]. This can be dealt with by collaborating with various partners to address the perceived needs of health facilities and communities at the level of the decentralized management units.

Communities, care providers, health facility managers, donors and the MOH each have important roles to play in establishing sustainable HIV programmes.

## Case description

### The project

In October 2004, FHI/ZPCT began work to strengthen and expand existing public sector, facility-based HIV/AIDS services in five of Zambia's nine provinces. The partnership, which operated until September 2009, was based on a cooperative agreement funded by PEPFAR through USAID. ZPCT worked within the Government of Zambia's existing structures, in collaboration with the MOH at all levels, to implement project strategies to initiate, improve and scale up PMTCT, counselling and testing and clinical care services for people living with HIV/AIDS, including ART programmes in target provinces. Interventions were tailored to follow MOH and the National HIV/AIDS/STI/TB Council policies and guidelines, as well as to adopt the national-level monitoring and evaluation system in line with the "Three Ones" principles [12]. A total of 219 health facilities in 35 districts were reached.

The Government of the Republic of Zambia's health system is decentralized, as in many African countries. The central MOH provides leadership and direction at the national level, with support from the provincial health offices. Within each province are districts, each of which has a district health office that oversees implementation of activities and supervises health facilities within its district. General and provincial hospitals have their own hospital management boards that report directly to the provincial health offices.

ZPCT-supported activities were targeted at hospital and clinics under the purview of the district health offices and the hospital management boards. To foster strong collaboration and partnership at the facility and district level, proposed activities were discussed after a joint assessment of facilities. Recipient agreements, signed between ZPCT and the district health offices and hospital management boards, detailed the scope of technical and programmatic activities to be conducted in supported health facilities at specified costs. This was in addition to umbrella agreements with provincial health offices and the MOH.

The project supported health facilities from May 2005 until July 2009, and provided HIV counselling and testing to 507,208 clients, PMTCT to 351,945 pregnant women, and ART to 84,973 clients, including 6153 children.

ZPCT II, a follow-on project, was awarded by USAID in June 2009 and is continuing to support the facilities. ZPCT technical support included training of healthcare workers and key district and provincial health office staff; this was done through MOH-approved training packages and standard operating procedures and provision of ongoing mentorship and supportive supervision of healthcare workers in facilities.

The partnership supported the MOH in the collection and documentation of all HIV/AIDS service statistics using the MOH health management information systems. The ZPCT-supported data-entry clerks assisted the facilities with routinely submitting data to the district, provincial and national levels. Facility-level statistics were available to assist facility staff with ongoing project monitoring.

Programmatic support was provided to strengthen health systems at district and provincial levels. Support included the renovations of facilities and procurement of essential equipment, including laboratory equipment. The capacity of the MOH was enhanced in the areas of logistics and commodity management systems, laboratory sample referral systems, patient referral networks, patient flow systems, service organization, and laboratory and pharmacy support services. These processes allowed skills to be transferred and consolidated among the healthcare workers, thereby enhancing their competencies.

A QA/QI system was developed to detect and address gaps in HIV service quality measured against established national standards. QA/QI skills were transferred to MOH staff at the levels of facilities and district and provincial health offices through formal orientation sessions for focal point persons providing HIV services. This was complemented by mentorship and facilitative supervision at health facilities during planned quarterly visits. Quality of services was monitored using standardized data collection tools; immediate feedback was provided to healthcare workers and district health office on priority areas identified as necessary for quality improvement [13].

Although the project showed that it was feasible to provide increased access to HIV interventions in resource-poor settings, the challenge of making them sustainable remains [2,6]. This paper presents an operational model based on achieving technical and programmatic sustainability in resource-poor countries, and discusses the process of transitioning technical support from a donor project to the MOH.

### Realizing operational sustainability: the ZPCT experience

The initial step taken towards realizing operational sustainability was to introduce a robust QA/QI system anchored in national standards that could be implemented by healthcare workers focused on HIV services. This presented a mechanism to measure and track quality gaps in a way that allowed healthcare workers to visualize, participate in and obtain immediate results that they could use to prioritize problems for remedial action.

The QA/QI provides a structured set of data collection tools, involving checklists, interviews by healthcare workers and patient record reviews. These tools assessed the "actual quality" of HIV technical and programmatic interventions and identified quality gaps at facility level for remedial action [13]. The tools enabled HIV service provision to be objectively assessed in identified key areas of operation required for successful service delivery within facilities delivering ART, clinical care, counselling and testing, PMTCT, pharmacy services, laboratory services, and monitoring and evaluation. These included the implementation of technical strategies, spatial service organization, human resource capacity and competence, information management, referral systems and commodity management.

For example, the tools ask: if healthcare workers attending to HIV patients in the ART clinic have received paediatric ART and opportunistic infection training based on the MOH package; if ART providers report any adverse drug reactions; if there is dedicated space for counselling and testing; and if charts to track expiry of drugs are available [13]. Information is also gathered by observation, staff interviews, patient file reviews (a minimum of 20 adult and 10 paediatric files per site are randomly selected) and register reviews. Notably, this information underpins service quality and was not captured by the health management information systems.

MOH personnel were oriented at provincial, district and health facility levels on the objectives and processes of the QA/QI system, including utilization of results obtained. This increased their buy in and enthusiasm for the process. Though an important pre-requisite for the success of the QA/QI process, it was not the only requirement for motivating facility healthcare workers to carry out QA/QI activities in the absence of direct financial support. Encouraging district health offices and facility management to participate in the process was crucial, particularly during the results and action planning sessions, where quality problems were outlined and remedial actions for quality improvement were identified jointly with facility healthcare workers. Motivation by healthcare workers to continue the QA/QI process was also reinforced through close collaboration with facility staff to recognize the benefits of obtaining QA/QI results, discussing results and progress in minimizing quality gaps, and tabling these QA/QI issues during facility- and district-level review meetings.

The QA/QI cycle is a systematic means of identifying a health facility's ability to implement quality services; it determines if the facility is ready to function without continuous project technical and programmatic support.

The next step towards attaining operational sustainability looked at each district's ability to offer effective HIV service management. Graduation criteria assessed were technical capacity, human resource management, commodity management and information management. Within each service area, there is a set of minimum standards. A different set of checklists, or graduation tools, were used to assess each district's ability to manage and supervise facility-based HIV programmes [14]. These graduation tools were applied at regular intervals in earmarked districts, where facilities had demonstrated good service delivery.

Remedial measures were instituted with project support in collaboration with the each district health office to improve indicator results until the overall district indicator scores met the required target. This status had to be maintained over three to six months in order for a district to "graduate" to the point where intensive programmatic and technical support was no longer provided.

**Figure 2:**
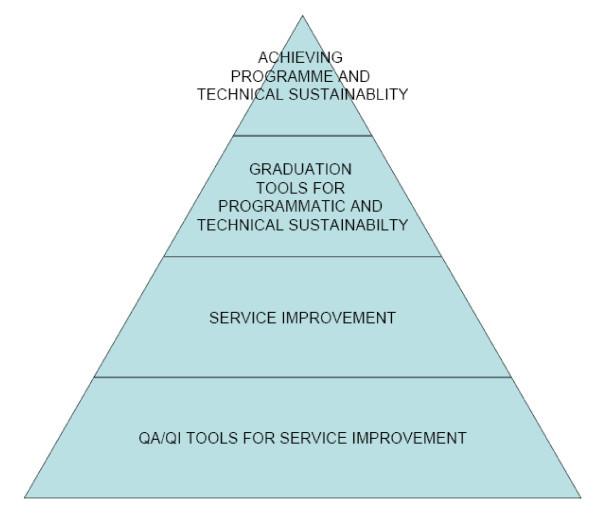
**Schema for realizing programmatic and technical sustainability**.

Supported by the ZPCT, 10 districts were successfully graduated from technical and programmatic intensive support. However, financial support was maintained for ongoing project implementation. Figure 2 illustrates the process.

The districts received an average of 40 months of project support (ranging from 32 to 48 months) before graduation. A percentage scoring system based on technical strategy implementation, commodity management, information management and human resource management was used to determine district readiness for graduation. The optimum score was 90% and above, defined as high service quality with low technical need. Earmarked districts were assessed over four consecutive quarters, and when their scores passed 90%, they were graduated. Table 1 shows the trends of scores after intense technical assistance prior to attainment of graduation status.

**Table 1:** Districts graduated from project support

	Overall score(%) by quarter					
District	Q3 2008	Q4 2008	Q1 2009	Q2 2009	Q3 2009	Months with project support before graduation
District 1	91.5	86	90	90	G	3 yr 10 mo
District 2	87	92.2	G	G	G	2 yr 11 mo
District 3	87	92.9	G	G	G	3 yr
District 4	97	G	G	G	G	3 yr 1 mo
District 5	84	92.4	G	G	G	3 yr 10 mo
District 6	78	85.4	92.6	G	G	3 yr 10 mo
District 7	87	87.8	93.2	G	G	3 yr 3 mo
District 8	89.6	84.2	94.2	G	G	3 yr 4 mo
District 9	83	84.4	94	94	G	4 yr
District 10	82.5	83.2	91.4	91.4	G	2 yr 8 mo
					Average	3 yr 4 mo
Key:	G = GRADUATED					

After graduation, financial support continued. However, intense technical assistance by the project was replaced with management plans, developed and implemented with provincial and district health offices as roadmaps to maintain technical and programmatic sustainability. This involved providing some technical assistance for district health offices and health facilities, where required, to maintain the continued delivery of high-quality services.

Challenges encountered during the graduation process included poor staff attitudes towards QA/QI prior to training, high turnover and perceived extra workload. Facility healthcare workers often perceived the quality and district graduation strategy as project owned and, as such, an extra duty. This also resulted in some healthcare workers expecting financial motivation in the form of remuneration to perform QA/QI and graduation activities, contrary to project policy. Several facility-level healthcare workers, as well as MOH officers at provincial and national level, exhibited keen interest in and enthusiasm towards the project's quality improvement and graduation sustainability strategy; they recognized its usefulness as an important results-based management tool.

## Discussion and evaluation

A number of technical approaches can be employed by donor-funded HIV projects to promote sustainability. HIV service provision is strengthened by enforcing the use of standardized national guidelines and standard operating procedures for HIV prevention, care and treatment, rather than instituting *ad hoc *project-driven interventions [15]. This allows for standardized, high-quality care across facilities receiving donor support. It encourages practices at service delivery points to be regarded as acceptable and MOH owned, and thereby likely to be continued, even in the absence of donor support. In addition to project staff providing training, technical support and mentorship based on national guidelines for HIV, establishing the QA/QI system in facilities further strengthens these guidelines. It creates and strengthens a platform for the MOH to continue providing technical support in the future.

Utilizing and strengthening already existing MOH supply and logistics management systems for procuring, distributing and maintaining HIV service-related equipment and commodities is essential. This includes following the MOH standards for laboratory equipment procurement to ensure standardized maintenance and reagent procurement [16]. Using the MOH-approved curricula to transfer skills in ART, clinical care, PMTCT, counselling and testing, and laboratory and pharmacy services is also essential [11]. Where non-existent, training packages developed by the project should be shared with the MOH and other partners to facilitate national adoption.

Introducing a QA/QI system specifically tailored towards HIV services provides a basis for continuous assessment and monitoring of HIV services. Standards used in this QA/QI system should be drawn from the MOH national standards, guidelines and standard operating procedures [14]. In circumstances where a QA/QI system is not an institutionalized component within the MOH's ambit, efforts to initiate this institutionalization is required. The MOH should be supported in adopting the tools, and it should formally lead the process.

Having an established QA/QI system for HIV services promotes technical sustainability through ongoing identification of quality gaps and implementation of remedial actions. It also raises awareness of healthcare workers of the importance of continuing to adhere to standards and good technical practices. It is necessary to integrate HIV support into already existing MOH health systems. This is central to strengthening the health system and preventing duplication of efforts. Furthermore, strengthening health systems contributes towards achieving sustainability [17].

A transparent exit strategy that transitions project support to the MOH needs to be developed early in the life of the project in order to prepare facilities, district and provincial health offices and the MOH to be systematically weaned off donor project support. This is achieved by establishing good working relationships between the project and MOH staff, based on mutual trust and respect. The MOH needs to be kept informed and participate fully at each step of the process. The exit strategy should be based on evidence of good quality services attained in supported districts. A series of objective, measurable and verifiable criteria through QA/QI and graduation tools needs to be applied to determine readiness of districts to be graduated from intensive project support [14].

The ZPCT experience indicates that it is feasible and practical to prepare districts for graduation from project support by starting with improving the quality of services at individual facilities and building up the districts' ability to manage the HIV services. It is the district health offices' role to ensure the continuous quality of health services. It is more practical to manage graduation at the district level, and permit reallocation of technical and programme resources within the districts as needed or to other districts. Graduated districts should not be left alone after achieving the desired quality levels, but should receive supportive supervision to maintain service quality and prevent retrogression. This is the role of the provincial health offices.

Sustainability of HIV services also depends on continued demand for services [8,11]. While social sustainability was not addressed in this paper, ZPCT did have a community component to create demand for HIV services. Potential obstacles may occur in achieving sustainable HIV services following withdrawal of project support. Lack of national institutionalized quality systems undermines the continuous implementation of the QA/QI approach on which the technical sustainability and exit strategy is based.

Despite training of MOH healthcare workers in specialized areas of HIV prevention, care and treatment, chronic understaffing of skilled health workers remains a challenge. Attrition, staff transfers and retirements have the potential to create a void if not appropriately planned and managed, negating positive gains in service quality.

Successful sites and districts have made efforts to minimize the effect of staff transfers and attrition through advocacy and planning. The willingness of management to embrace the graduation concept and subsequently motivate health workers is another beneficial characteristic of successfully graduated sites. Poor physical infrastructure may negatively impact on the graduation process as a result of its effect on technical sustainability.

In the absence of pre-service and in-service introduction to QA/QI concepts, introducing this concept through a project arouses some resistance and suspicion of "policing" among healthcare workers at facility level. Ongoing mentorship and supportive supervision is a necessary part of the change management that is required to build capacity and maintain understanding and demand for the QA/QI process. In the absence of strong leadership at district level to motivate and drive facility-based QA/QI initiatives, there is a risk of QA/QI activities waning.

Inability to guarantee continuity of future sources of funding, particularly donor funds, is a source of valid concern in government budget planning for sustained HIV services [18].

Implications exist for the MOH and development organizations to facilitate sustainable HIV services. As HIV services are scaled up, there is a need to develop, implement and institutionalize a standardized QA/QI system for HIV services. These should be applied uniformly across all HIV prevention, care and treatment services, irrespective of the source of funding or support.

While our experiences present an important step towards technical and programmatic sustainability, the absence of financial resources, either internally generated or donor supported, can be a limitation. Furthermore, leadership at the decentralized management units of the healthcare system is necessary for success through providing strategic direction, planning and implementation.

## Conclusions

Achieving operational sustainability in a resource-limited setting is practical and feasible. Developing and institutionalizing a quality assurance/quality improvement system is the basis of attaining graduation and sustainability of services. Use of national standards, guidelines, existing health system structures, logistics and information management are vital for ensuring sustainability.

## Competing interests

The authors declare that they have no competing interests.

## Authors' contributions

KT and LM conceptualized the paper. KT, LM, CT, EM and WvD drafted and provided critical review of the manuscript. The views expressed in this article do not necessarily reflect those of FHI. The sponsor of the study had no role in the design, data collection, analysis, interpretation and writing of the report. The corresponding author had full access to data and had final responsibility for publishing this paper. All authors read and approved the final manuscript.
